# Induced Sputum Is Safe and Well-Tolerated for TB Diagnosis in a Resource-Poor Primary Healthcare Setting

**DOI:** 10.4269/ajtmh.14-0583

**Published:** 2015-03-04

**Authors:** Cesar Ugarte-Gil, Paul T. Elkington, Eduardo Gotuzzo, Jon S. Friedland, David A. J. Moore

**Affiliations:** Instituto de Medicina Tropical Alexander Von Humboldt, Universidad Peruana Cayetano Heredia, Lima, Perú; Department of Infectious Diseases and Immunity, Imperial College London, London, United Kingdom; Clinical and Experimental Sciences, University of Southampton, Southampton, United Kingdom; Laboratorios de Investigación y Desarrollo, Universidad Peruana Cayetano Heredia, Lima, Perú; London School of Hygiene and Tropical Medicine Tuberculosis Centre and Department of Clinical Research, London School of Hygiene and Tropical Medicine, London, United Kingdom

## Abstract

Improved tuberculosis (TB) diagnostics are required. Induced sputum sampling is superior to spontaneous sputum analysis for diagnosis of pulmonary TB. Therefore, we examined the applicability of induced sputum in primary health centers of the Peruvian TB program and studied the safety and tolerability of this procedure. We show that induced sputum is safe, inexpensive, and well-tolerated in a resource-limited environment. Widespread use of induced sputum at primary health centers can be implemented and may improve TB diagnosis.

## Introduction

Tuberculosis (TB) remains a major diagnostic challenge in resource-poor settings, despite the global effort in developing new diagnostic tests and biomarkers. Failure to diagnose pulmonary TB early in the course of infection results in progressive lung disease, cavitation, and ongoing transmission of infection, because patients with pulmonary cavities are highly infectious. A fundamental problem is the low sensitivity of spontaneous sputum smear for acid-fast bacilli (AFB) to diagnose pulmonary TB.^1^ One central reason for this is the highly variable quality of sputum samples: in many cases, patients need to produce two or three samples for diagnosis.^2^

The induced sputum procedure is a well-established technique^3^ to obtain consistent samples from the respiratory tract.^4^ Studies in TB have shown its efficacy in improving diagnosis, especially in children and human immunodeficiency virus (HIV) -positive patients.^4^ However, the use of induced sputum is primarily restricted to tertiary-level health centers. The potential of induced sputum as a method to obtain improved sputum samples for research purposes in primary healthcare settings, where the majority of TB patients access care, has recently been shown.^5^–^7^ Therefore, we studied the tolerability of induced sputum for diagnosis of TB in a resource-poor primary healthcare setting and show that induced sputum is safe and well-tolerated in patients with TB.

## Methods

Participants were recruited for a cross-sectional and cohort study among patients presenting with symptoms suggestive of pulmonary TB. The inclusion and exclusion criteria have been described previously.^8^ Sixty-eight adult TB patients were recruited prospectively at the time of diagnosis with a smear and/or culture positive for *Mycobacterium tuberculosis*. Sixty-nine healthy controls were recruited in the same setting as TB patients, and they had sputum smears and cultures negative for *M. tuberculosis*.

To study the safety of the procedure, a research nurse recorded the O_2_ saturation with an NBP-40 saturometer (Nellcor Puritan Bennet, Galway, Connacht, Ireland), blood pressure (at the beginning and the end of the procedure), and any adverse events that occurred. We considered O_2_ saturation below 92% as hypoxemia.^9^

Induced sputum was performed with a portable compressor nebulizer NA180 (Aspen, Buenos Aires, Argentina) with a disposable mask in a specially designed room at the Tuberculosis Clinic without a roof, with direct sunlight, and good air flow for good ventilation. The nebulizer cost is $90, and disposables were $4 for each patient (saline, mask, and disposable tubing). The procedure took a total of 30 minutes and required 10 mL 3% saline (Farmacia Universal, Lima, Peru) using a similar protocol described before.^8^,^10^ The analysis was done with Stata 12 (StataCorp, College Station, TX). This study was approved by the Ethics Committee of the Universidad Peruana Cayetano Heredia.

## Results and Discussion

There were 137 participants: 69 (50.4%) healthy controls and 68 (49.6%) TB patients. The characteristics of the population and sputum samples are shown in Table 1. The procedure was well-tolerated. Among healthy controls, only dizziness was reported as an adverse event in two (3%) participants. Among TB patients, minor events reported were nausea, headache, tachycardia, and dyspnea (three participants for each adverse event; 4.4%): one participant reported experiencing all four of these adverse events, one participant reported two events (tachycardia and dyspnea), and six participants reported only one event. All participants completed the procedure without any serious complications. Mean O_2_ saturation at the beginning and the end of the procedure was similar and within safe ranges, and there was no difference between TB patients and healthy controls (Table 1). All symptoms were mild, and no additional interventions beyond reassurance and comfort measures were required. All symptoms disappeared within 20 minutes of the procedure. There were no associations between TB status, body mass index (BMI), and age at enrollment with the likelihood of experiencing an adverse event.

Induced sputum has previously been shown to be a safe procedure in the case of asthmatic patients, with good tolerability and minimum clinical risks.^11^ Also, this procedure showed an increased sensitivity for TB diagnosis in patients with problems producing spontaneous sputum samples (such as HIV-positive patients and children).^12^ Similar to our study, good tolerance of the induced sputum procedure was seen among adults and adolescents with suspected TB (HIV-positive and -negative) in a community healthcare setting in South Africa.^6^ We did not perform a pre-nebulization step and still found no significant adverse events, showing that induction directly with hypertonic saline can be undertaken safely. Additionally, we reveal no difference in the likelihood of adverse events between healthy controls and TB patients.

Improved diagnostic tests for pulmonary TB are urgently required. Sputum induction is relatively easy to perform at the primary health level and well-tolerated in open-air conditions. Some studies refer to a potential risk for nosocomial transmission^13^,^14^; however, the design of the room (with open air and direct sunlight) reduces this potential risk. Adequate natural ventilation showed efficiency in nosocomial settings,^15^ and in addition to adequate biosafety training, protection measures for the personnel (i.e., N95 masks) should be the norm in all sputum procedures (induced sputum, smear, and culture procedures) The sensitivity, tolerability, and low cost of induced sputum make it a powerful tool in low-resource settings to improve the quality of sputum samples. The advantages of induced sputum are the simplicity of the technique and its use for both diagnostics and research; in our study, we used an inexpensive but robust electrical machine, which can be used in resource-poor settings. The cost of $4 for the kit per patient can be cost-effective, considering that the delay in diagnosis increases the risk of medical complications and transmission with the consequent increases in the costs for the patient and the health system.

This study has some limitations. We did not measure forced expiratory volume (FEV) to assess potential airway constriction, but it is difficult to measure FEV in this setting because of concerns relating to infection control (disposable materials, etc.), which is not normally available in low-income settings. Considering that, in both studies in resource-poor settings, none of the participants had severe adverse events after the procedure, we can assume that induced sputum poses minimal risk for airway constriction. Nevertheless, we suggest first evaluating the risk for airway constriction (low O_2_ saturation, previous history of asthma attacks, or other conditions for airway constriction) and second, having an emergency kit, including salbutamol for nebulization, available (which should be available in any healthcare center) to reduce this potential risk.

One limitation of induced sputum for routine diagnosis is that, on average, the procedure takes 30 minutes. In a setting with a high flow of patients, this would be problematic, and therefore, induced sputum should be reserved for diagnostic challenges, such as children and patients with non-productive cough, like in early pulmonary TB. However, its simplicity makes this procedure a viable solution in settings where there is a shortage of doctors, and it can be performed by nurses and health technicians.

In conclusion, induced sputum is a safe procedure in primary healthcare settings and can be deployed to improve the quality of sputum for diagnosis or research purposes. For non-productive respiratory symptomatic or HIV-positive patients, wider use of induced sputum may greatly increase TB diagnostic yield without risk of adverse events.

## Figures and Tables

**Table 1:** Characteristics of the participants

Characteristics	Healthy participants	TB participants	*P* value*
Male *N* (%)	12 (17.4)	42 (61.8)	< 0.005
Age (years) mean (SD)	31.7 (1.5)	31.1 (1.7)	0.48
BMI mean (SD)	25 (0.5)	21 (0.4)	< 0.005
Axillary temperature mean (SD)	36.2 (0.02)	36.6 (0.07)	< 0.005
Heartbeat mean (SD)	75.4 (1.1)	94.4 (1.8)	< 0.005
Saturated O_2_ before induced sputum mean (SD)	98.6 (0.1)	97.8 (0.2)	< 0.005
Saturated O_2_ after induced sputum mean (SD)	98.9 (0.1)	98.2 (0.2)	< 0.005
Systolic blood pressure before induced sputum mean (SD)	113.1 (0.9)	111.5 (1)	0.21
Systolic blood pressure after induced sputum mean (SD)	112.1 (1.2)	113.9 (1.2)	0.29
Sputum volume mean (SD)	6.4 (0.4)	5.9 (0.3)	0.32

* *P* value for *t* or χ^2^ test to assess differences between healthy and TB participants.
